# Exploring rural middle school music teachers’ classroom interaction decision-making levels: A student’s perspective

**DOI:** 10.1038/s41598-025-90503-4

**Published:** 2025-02-17

**Authors:** Chulan Xue, Zongchen Hou, Ruicong Ma

**Affiliations:** 1 Faculty of Music & Performing Arts, Universiti Pendidikan Sultan Idris, Tanjong Malim, Malaysia 35900; 2 School of Music, Shandong Women’s University, Jinan, China 250300

**Keywords:** Perspective of students, Teaching decision-making level, Rural middle school, Music education, Psychology, Human behaviour

## Abstract

This study explores students’ perceptions of rural middle school music teachers’ decision-making levels in the context of classroom interactions. Using a questionnaire survey of 246 students from a rural public middle school and follow-up interviews, we investigate students’ overall perceptions and examine differences among students of different genders, grades, and music study experiences. The results indicate that students generally perceive teachers’ decision-making levels to be slightly above average, with no significant differences between male and female students or among different grade levels in their perceptions of teacher decision-making levels. However, students with and without music study experience differ significantly in their perceptions of teacher decision-making levels. The qualitative insights gathered from the student interviews complement the quantitative findings, providing a deeper understanding of students’ perspectives on teacher decision-making in rural music education. Overall, this study provides valuable insights for enhancing the educational practices of teachers and explores effective teaching strategies in rural environments.

**Supplementary Information:**

The online version contains supplementary material available at 10.1038/s41598-025-90503-4.

## Introduction

In middle school music education, classroom interaction plays a crucial role, especially in resource-constrained environments such as rural areas. The decisions made by music teachers during these interactions directly impact students’ learning experiences and performance^1^. Understanding and evaluating these decision-making levels is essential, as they significantly influence students’ engagement and overall learning outcomes^2^. The teaching decision-making ability of music teachers significantly influences students’ music study experiences and achievements^3^. However, in rural areas, music teachers often encounter complex decisions regarding teacher-student interactions, classroom management, adaptability, and teaching skills, which not only shape students’ learning but also influence teachers’ professional growth^4,5^.

Despite the undeniable importance of music education, there is a lack of existing research focusing on rural music education decision-making, particularly from the perspective of students in evaluating teachers’ decision-making levels^6^. Additionally, rural schools face limited resources and a shortage of specialized music teachers, further highlighting the need to investigate the unique characteristics of rural music education^7^. The goal of this study is to explore rural middle school music teachers’ classroom interaction decision-making levels from the students’ perspective, address this research gap and provide theoretical and practical guidance to improve educational quality.

Furthermore, students, as direct participants in the teaching process, provide valuable, experience-based feedback that reflects the effectiveness of teaching. Their feedback helps teachers better understand students’ needs, fostering reflection and continuous improvement in teaching strategies. By incorporating students’ perspectives, teachers can adjust methods to better meet learning requirements, contributing to sustained improvements in educational quality.

This study intends to investigate the following research questions:

1. How do students perceive music teachers’ decision-making levels?
2. How do these perceptions vary by gender, grade, and music study experience?

## Literature review

The literature review focuses on three key areas: student perspectives in educational research, teaching decision-making theory, and the unique context of rural music education. Classroom interaction is a critical factor in promoting student learning, with teachers’ decisions playing a decisive role. This study investigates the teaching decision-making levels of rural secondary school music teachers from the students’ perspective, aiming to provide practical recommendations for improving rural music education.

### Educational research from a student perspective

Student-centered educational research has gained increasing prominence in Chinese education, reflecting a shift toward prioritizing students at the core of teaching^4^. Students’ perceptions are recognized as essential for evaluating teaching quality, offering valuable insights into their experiences, needs, and learning challenges^8^. This perspective enables educators to refine teaching strategies based on student feedback, which aligns with their actual learning conditions and preferences.

Internationally, research underscores the benefits of integrating student perspectives to enhance engagement and academic outcomes. Recent studies have emphasized the impact of classroom dynamics on study experiences and the importance of incorporating student voices into decision-making processes to improve motivation and achievement^8^. This approach is particularly valuable in rural education, where addressing students’ unique needs and expectations in resource-constrained environments can significantly enhance learning outcomes^6,9^.

Recent studies further stress the role of digital tools in capturing real-time student feedback, fostering more responsive teaching practices^9^. This aligns with the growing emphasis on data-driven educational strategies, particularly in under-resourced rural areas^10^.

Building on previous research, this study examines the cognitive differences among students of different genders, grades, and musical backgrounds, contributing to the development of targeted and effective teaching strategies. The incorporation of recent international perspectives ensures a broader and more relevant framework for analyzing rural music education contexts.

## Teaching decision-making theory

Teaching decision-making consists of three stages: pre-active, interactive, and post-active^11^. Research consistently highlights the interactive stage as crucial for fostering student engagement and learning^12^. Effective classroom interaction enhances focus and deepens musical understanding, whereas poor decision-making can hinder educational outcomes^13^.

Recent studies emphasize the importance of teacher adaptability and classroom management in the decision-making process^14^. Factors such as teaching skills, the classroom environment, and feedback mechanisms directly influence interactive decision-making^15^. In rural music education, limited resources and classroom dynamics further shape these decisions, requiring a flexible and responsive approach^1^.

With the introduction of new music curriculum standards in China, the development of teaching decision-making skills has become an academic focus^8^. Studies indicate that teaching decisions not only guide student learning but also influence academic performance and interest^6^. This study extends existing literature by exploring how rural teachers’ decision-making processes adapt to real-time classroom challenges, contributing to a more comprehensive understanding of interactive teaching decision-making.

Despite the widespread application of teaching decision-making theory in educational research, few studies have focused on rural music education, particularly regarding how student feedback reflects teachers’ interactive decision-making levels. This study aims to address this gap by exploring students’ perceptions of teachers’ classroom interactive decision-making and providing empirical evidence to improve rural music education.

## Conceptual framework

**Fig. 1:**
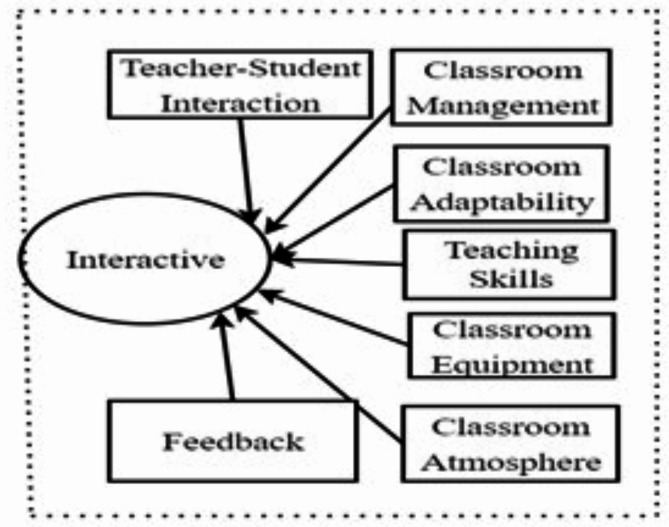
Factors Influencing Interaction Decision-Making.

The conceptual framework of this study is based on the three-stage theory of teaching decision-making (pre-active, interactive, and post-active stages)^11^and research on teachers’ classroom interactive decision-making^10^ (see Fig. 1). Classroom interaction is a crucial component of the teaching process and directly affects students’ study experiences and knowledge retention^5^. Effective classroom interaction can stimulate students’ interest, enhance classroom engagement, and deepen their understanding and application of musical knowledge^1^.

The conceptual framework guided data collection by shaping the design of the questionnaire and interviewing questions, with a focus on the following:

1. Teacher-Student Interaction: Positive teacher-student interaction effectively promotes student learning, including verbal communication, answering questions, and encouraging participation in classroom activities^12^.
2. Classroom Management: Effective organization and control of student behavior by teachers contribute to creating a positive learning environment, minimizing disruptive behavior, and enhancing learning efficiency^8^.
3. Classroom Adaptability: Teachers’ ability to respond flexibly to unforeseen situations or students’ learning difficulties enhances teaching effectiveness^6^.
4. Teaching Skills: Teachers’ professional skills and teaching methods directly influence the quality of classroom interaction and student engagement^3^.
5. Classroom Equipment: The adequacy and modernization of teaching equipment play crucial roles in shaping students’ learning experiences and the attractiveness of course^16^.
6. Classroom Atmosphere: A positive classroom atmosphere encourages active student participation and deeper thinking, further stimulating interest in learning^3^.
7. Feedback: Timely and specific feedback mechanisms help students understand their learning progress and adjust their learning strategies accordingly^5^.

**Fig. 2:**
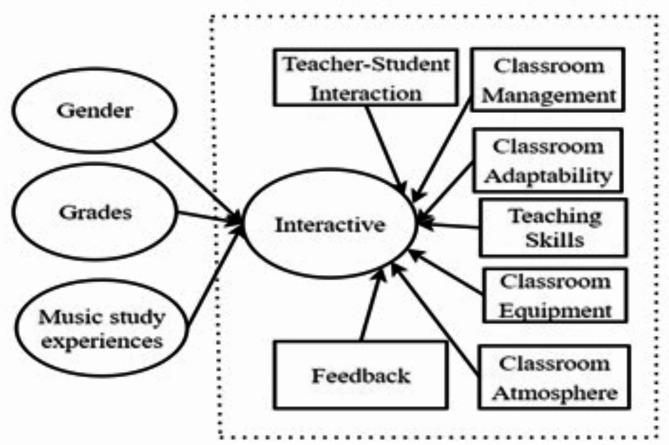
Model of Factors Influencing Interaction Decision-Making.

This study is based on the students’ perspective, and therefore, it is crucial to explore whether students’ personal factors further influence their perceptions of teachers’ interactive decision-making levels. Based on previous related research, this study identifies the following three factors that may influence students’ perceptions of teachers’ interactive decision-making (see Fig. 2).

Gender: Gender differences may lead to variations in students’ performance and participation in classroom interactions^13^. Studies have shown that female students typically exhibit higher levels of engagement and emotional investment in music classes, whereas male students may focus more on instrumental or rhythm-based activities. Such differences may influence how students perceive and evaluate teachers’ classroom interaction and management^1^.

Grade Level: Students at different grade levels have varying needs with respect to teaching methods and the classroom atmosphere. Lower-grade students require more direct guidance and support from teachers, whereas higher-grade students may prefer independent learning and critical thinking^17^. As a result, students’ perceptions of teachers’ interactive decision-making levels may change as their grade level increases.

Music Study Experience: Prior music study experience directly affects students’ understanding of the lesson content and their level of participation^14,15^. Students with music study experience may find it easier to engage in material and classroom interactions, whereas students without music study experience may feel pressured or disconnected during classroom activities, thereby influencing their evaluation of teachers’ interactive decision-making levels.

## Methodology

### Research design

The cross-sectional design was chosen because it efficiently captures diverse perspectives within a limited timeframe, aligning with the study’s goal of identifying patterns and differences without the need for longitudinal data collection^18^. This approach is particularly suitable given the constraints of working with a defined sample size in a rural educational setting.

### Data collection instruments

Data Data collection occurred in two phases. First, a structured questionnaire was electronically distributed via QQ, WeChat, and email to all 356 students, resulting in 246 valid responses (69.1% response rate). Participation was voluntary, allowing students to respond at their convenience without disrupting school activities.

In the second phase, semi-structured interviews were conducted with three randomly selected students to gain deeper qualitative insights into their perceptions of music teachers’ classroom decision-making.

SPSSAU software was used for data analysis because of its user-friendly interface and automated statistical tools, ensuring efficient and accurate processing of the survey data. This streamlined approach facilitated the handling of datasets in educational research^6^.

### Sample

1) Questionnaire Sample.

The study participants included all enrolled students from grades seven, eight, and nine in a rural public middle school in Jinan. The total number of students in the school is 356. The sample size was calculated as follows.

The margin of error was set at 4% with a 95% confidence level, ensuring that if 50% of the sample chose a particular response, the true population proportion would lie between 46% and 54%. A 95% confidence level strikes a balance between reliability and resource efficiency, avoiding excessive costs while ensuring dependable results^10^.

Sample Size Formula:

1 $$\:n=\frac{{Z}^{2}\cdot\:p\cdot\:(1-p)}{{E}^{2}}n$$

n = required sample size.

Z = 1.96 (for 95% confidence).

*p* = 0.5 (estimated success proportion).

E = 0.04 (margin of error).

Calculation:

2 $$\:{n}_{0}=\frac{{1.96}^{2}\times\:0.5\times\:(<span class=^{\prime} \, convertEndash^{\prime}\, >1-0.5</span>)}{{0.05}^{2}}=600.25$$

Rounded to 600.

Finite Population Correction (FPC):

Since the population size is 356, FPC is applied:

3 $$\:n=\frac{{n}_{0}\times\:N}{{n}_{0}+N-1}=\frac{600\times\:356}{600+<span class=^{\prime} \, convertEndash^{\prime} \, >356-1</span>}\approx\:223$$

Thus, the final sample size is 223.

The minimum required number of questionnaires for this study was 223. A total of 246 questionnaires were collected, which exceeded the requirements and ensured sufficient data for analysis.

2) Semi-structured interviews Sample.

This study selected three students to conduct semi-structured interviews. Three students were selected to conduct semi structured interviews based on:

i. Characteristics of Qualitative Research: The core of qualitative interviews lies in depth and detail, rather than in the breadth of the sample size^19^. Through a small number of participants, individual real experiences and specific feelings can be explored in depth, obtaining more layered and detailed descriptive data. Compared with large-scale surveys, qualitative interviews focus more on the diversity and typicality of viewpoints rather than the representativeness of data.
ii. Data Saturation Principle: In qualitative research, the principle of data saturation is generally followed, meaning that the sample size is considered sufficient when new interviews no longer generate new themes or viewpoints^20^. During the preliminary interviews, it was found that the responses of three students covered the main issues related to teaching interactions, classroom management, and feedback, with no significant new insights appearing. Therefore, interviewing three students was deemed sufficient to achieve the research objectives.
iii. Representativeness and Diversity: Although the number of interviewees was small, the research team ensured that the data reflected the views and experiences of students from different grades (one from each of grades 7, 8, and 9). This allows for a horizontal comparison of differences between grades, while also obtaining an overall impression of the teacher’s performance across different grade-level classrooms.
iv. Practicality and Feasibility: Considering the research resources and time constraints, large-scale interviews were difficult to implement. The selection of three students for interviews not only ensured the efficiency of data collection but also guaranteed the controllability of the research progress. Additionally, students in rural areas often face heavy academic pressure, and reducing the number of interviewees helps minimize disruption to their daily learning.
v. Supplementary Quantitative Data: The purpose of the interviews is to supplement the quantitative questionnaire data, providing richer background information and explanations^10^. Since the questionnaire already covered 246 students, the semi-structured interviews are intended to offer a more in-depth individual case analysis of the general trends reflected in the questionnaire. Therefore, the interview sample does not need to be large.

## Questionnaire design

This research adopted a cross-sectional survey design to understand the teaching decision-making levels of music teachers in a rural middle school in Jinan from the perspective of students. The study also aims to analyse whether there are differences in the perceptions of teacher teaching decision-making levels among students of different genders, grades, and music study experience.

The research used a structured survey questionnaire as the data collection tool. The questionnaire, consisting of 21 questions, required participants to answer based on their actual experiences and opinions. The questionnaire used a 5-point Likert scale, where 1 = strongly disagree and 5 = strongly agree. The questionnaire design involved the following steps:

Step 1: Draw from existing research. In exploring “teacher teaching decision-making,” valuable insights and theoretical foundations from existing studies were found. Previous research in the field has identified reference dimensions for scales that help understand and quantify teachers’ levels of decision-making in classroom interactions. For example, research has been conducted on mathematics education, and a questionnaire has been developed to assess music teachers’ decision-making^6^. These established scale dimensions served as the starting point for considering how to measure teaching decisions. Additionally, researchers have identified measurement dimensions and factors for decision indicators based on their research theories and specific content^1,5,6,10,11^. Their work provided insights into constructing teaching decision scales, resulting in an initial question bank comprising 21 questions.

Step 2: Expert validation. In a preliminary expert validation conducted in February 2024, five doctoral advisors with expertise in the field were invited. Detailed discussions were held regarding the logic of questionnaire dimension division, the appropriateness of content for each dimension, language accuracy and standardization, and potential issues in future data analysis. These discussions yielded valuable suggestions for modifications aimed at enhancing the quality and credibility of the survey.

Step 3: Pilot test. To assess the effectiveness of the research tools, a pilot test was conducted before the formal study to ensure the feasibility, reliability, and validity of the questionnaire. As noted by Song, pilot studies typically involve 30 to 50 participants. In this study, 60 questionnaires were distributed, with 54 responses received, resulting in a response rate of 90%. After incomplete responses were excluded, 48 valid questionnaires were used for analysis, with an effective response rate of 80%. Additionally, the data successfully passed Exploratory Factor Analysis (EFA), further validating the construct validity of the questionnaire. These steps ensured that the research design and data collection instruments were reliable and appropriate for the formal study.

Through these three steps, we refined the initial questionnaire, focusing on adjusting the question structure and fine-tuning the language to ensure better understanding by the students.

## Semi-structured interviews design

Based on the research objectives and the seven core factors, the following interview questions are designed for each factor. A semi-structured interview approach is adopted to balance consistency and adaptability, allowing respondents to reflect deeply while providing comprehensive and detailed information. This approach is widely recognized for its ability to explore participants’ perspectives while maintaining a structured framework^10^.

For example:

When you encounter difficulties in class, how does your teacher usually assist you?

What equipment or tools do you find most helpful in understanding the teacher’s explanations?

How does your teacher maintain discipline and order in the classroom? Do you think this management approach is effective?

The semi-structured interview data in this study underwent a systematic coding process to extract key themes and patterns, ensuring the scientific rigor and reliability of the results. The following outlines the coding process:

(1) Data Preparation.

The interview recordings were transcribed verbatim to ensure completeness and accuracy, minimizing the risk of missing important information. The transcripts were formatted uniformly, with clear labeling of questions and responses to maintain consistency. All the interviewees were anonymized (e.g., S1, S2) to protect their privacy and ensure confidentiality. Researchers then read and reviewed the transcripts multiple times to become thoroughly familiar with the content. During this process, preliminary notes were taken, highlighting key terms, themes, or patterns that emerged for further analysis.

(2) Initial Coding (Open Coding).

Granularity of coding involves breaking down the transcripts into smaller units, such as sentences or paragraphs. Each unit was assigned an initial code reflecting its core meaning (see Table 1). Descriptive codes were used to capture the explicit content of the participants’ responses. For example, if a participant said, “Group discussions help me understand musical concepts better,” it was coded as “Classroom interaction enhances understanding.”

**Table 1:** Examples of initial coding.

Interview Excerpt	Initial Code
The teacher often asks questions to engage us.	Classroom interaction, active questioning
Multimedia tools help me understand complex ideas.	Equipment-assisted learning, multimedia use
I concentrate better when the class atmosphere is relaxed.	Learning environment, relaxed atmosphere
The teacher provides detailed feedback on assignments.	Individual feedback, post-class guidance

(3) Categorization and Axial Coding.

A. Categorization.

Similar codes were grouped into broader categories that correspond to the seven core factors identified in the study. These categories helped organize the data for further analysis.

B. Axial Coding.

Based on the research framework, the coding process encompassed seven core factors: Teacher-student interaction; Classroom equipment; Classroom management; Classroom adaptability; Teaching skills; Classroom atmosphere; Feedback. Table 2 provides an example of code categorization.

**Table 2:** Example of Code categorization.

Core Factor	Sub-category	Code Example
Teacher-student interaction	Classroom engagement	“The teacher encourages questions”
Classroom equipment	Multimedia tools	“Videos help explain abstract ideas”
Classroom management	Discipline maintenance	“The teacher enforces submission deadlines”
Classroom atmosphere	Relaxed environment	“I focus better on a relaxed setting”

(4) Code Validation and Refinement.

A. Dual Coding and Cross-validation:

Dual Coding involves two researchers independently coding the same interview data. Comparison ensures coding consistency by comparing results, discussing discrepancies, and resolving any differences.

B. Expert Review:

The coded data were submitted to three experts in the field of education for review. The experts assessed the coding for accuracy, ensuring alignment with the study objectives.

### Reliability and validity

In this study, reliability refers to the consistency in measuring the expected attributes, associated with internal consistency^16^. The author evaluated it via Cronbach’s Alpha in Statistical Product and Service Software Automatically (SPSSAU).

The Cronbach’s Alpha values for each subscale were as follows: Teacher-student interaction (α = 0.931), Classroom management (α = 0.838), Classroom adaptability (α = 0.885), Teaching skills (α = 0.848), Classroom equipment (α = 0.929), Classroom atmosphere (α = 0.730), and Feedback (α = 0.935). The overall Cronbach’s Alpha was 0.926. All Alpha values were relatively high, indicating strong internal consistency within the respective subscales. This enhances the reliability of the measurement tool in assessing the target constructs in the study. Furthermore, the corrected item-total correlations (CITC) exceeded 0.4, indicating strong correlations among the analysed items, thus demonstrating high reliability.

Validity: Further confirmation of the validation was obtained, with a KMO value of 0.94, exceeding the threshold of 0.8, and a p-value for Bartlett’s test below 0.05. These results demonstrate the effectiveness of our research tool.

### Ethical approval

Ethical research necessitates expertise, diligence, and steadfast honesty to protect the rights of human subjects. Upholding principles such as self-determination, anonymity, confidentiality, and informed consent is paramount^18^. Prior to participation, oral permission was obtained from the staff and students at the middle schools involved. The participants provided informed consent, ensuring that they were fully briefed on the study’s purpose and data collection procedures and that they were assured of the absence of potential risks or costs. To ensure participants’ anonymity and confidentiality, all identifiable information was removed from the data, and electronic survey responses were securely stored and password-protected. Additionally, participants were informed that they could withdraw from the study at any time without any negative consequences. The study was conducted in accordance with the Declaration of Helsinki, and the protocol was approved by the Institutional Review Board of Universiti Pendidikan Sultan Idris (UPSI). The reference number for ethical approval is: 2024-0384-01. For the interviews, three students were chosen based on purposive sampling criteria, ensuring that they represented a range of perspectives and experiences relevant to the study’s objectives. The selection was made by a pool of volunteer participants from the same school, allowing for a deeper understanding of the specific context and dynamics within that educational environment.

## Results

### Question 1

Table 3 presents descriptive statistics reflecting students’ positive attitudes toward teachers’ decision-making in classroom interactions. The overall mean score for teaching decision-making was 3.310 (SD = 0.714), with a median of 3.410. Among the seven factors, classroom adaptability had the highest mean score (M = 3.541, SD = 1.069), followed by classroom equipment (M = 3.515, SD = 1.077). The lowest mean scores were observed for the classroom atmosphere (M = 3.049, SD = 1.147) and feedback (M = 3.050, SD = 1.100).

**Table 3:** Descriptive analysis.

Items	*N* of samples	Min	Max	Mean	Std. Deviation	Median
Overall	246	1.390	4.950	3.310	0.714	3.410
Classroom equipment	246	1.000	5.000	3.515	1.077	3.600
Classroom management	246	1.000	5.000	3.472	1.083	3.600
Classroom adaptability	246	1.000	5.000	3.541	1.069	3.600
Teaching skills	246	1.000	5.000	3.366	1.102	3.500
Teacher-student interaction	246	1.140	5.000	3.374	1.048	3.570
Classroom atmosphere	246	1.000	5.000	3.049	1.147	3.130
Feedback	246	1.000	5.000	3.050	1.100	3.200

### Question 2

Although Research Question 2 explores students’ perceptions of teachers’ decision-making levels, it involves three key variables: gender, grade level, and music study experience. To present the data analysis process and research findings more clearly, this study divides the analysis of Research Question 2 into three separate sections. This approach not only enhances the readability of the data but also ensures alignment between quantitative and qualitative data, making the research findings more targeted and interpretable.

(1) Different Genders.

Table 4 shows the nonparametric statistical analysis of students’ perceptions of teachers’ decision-making levels across different genders. The overall median scores for boys and girls were 3.410 and 3.380, respectively, with no significant difference observed (*p* = 0.626). Similarly, no statistically significant differences were found across six dimensions, including classroom equipment, classroom management, classroom adaptability, teaching skills, classroom atmosphere, and feedback (*p* > 0.05). However, a significant difference was noted in the dimension of teacher-student interaction (*p* = 0.014), indicating that boys perceived higher levels of interaction (Median = 3.710) than girls did (Median = 3.290).

**Table 4:** Nonparametric Statistical Analysis Table for students of different genders.

	Gender Median (*P*_25_, *P*_75_)		MannWhitney U	MannWhitney z	*p*
	Girl (*n* = 135)	Boy (*n* = 111)			
Overall	3.380(2.9,3.8)	3.410(2.9,3.9)	7221.500	−0.488	0.626
Teacher-student interaction	3.290(2.1,4.1)	3.710(2.9,4.1)	6132.000	−2.452	0.014*
Classroom equipment	3.600(2.6,4.4)	3.600(2.8,4.4)	7455.500	−0.067	0.947
Classroom management	3.600(2.8,4.4)	3.400(2.6,4.0)	6804.500	−1.241	0.214
Classroom adaptability	3.800(3.0,4.6)	3.400(2.6,4.2)	6460.500	−1.863	0.062
Teaching skills	3.330(2.3,4.2)	3.500(2.8,4.3)	6558.000	−1.685	0.092
Classroom atmosphere	3.130(2.0,4.0)	3.130(2.3,3.9)	7373.500	−0.214	0.830
Feedback	3.200(2.2,4.0)	3.000(2.2,3.8)	7170.000	−0.582	0.561

* *p* < 0.05 ** *p* < 0.01.

The students’ responses further validate the conclusion: Student 1: “I feel that both male and female teachers handle classroom interactions well. They seem to make decisions that keep the class running smoothly. I have not noticed any significant differences in classroom management based on gender.” Student 2: “I agree. Our teachers, regardless of gender, seem to manage the classroom effectively. They adjust their teaching styles and interact with students to facilitate learning. I have not observed any significant gender-based differences in teaching methods.” Student 3: “I have also noticed that both male and female teachers make decisions in class that help us learn. They interact with students and create a positive learning environment. I have not seen any significant differences in their interaction styles based on gender.” These responses further support the conclusions presented in Table 4, indicating that there is no significant difference in the perceptions of music teachers’ interaction decision-making levels among students of different genders.

(2) Different Grades.

Table 5 presents the nonparametric analysis of students’ perceptions of teachers’ decision-making levels across different grades. The results indicate no statistically significant differences in overall perception (*p* = 0.844) or in any of the subcategories, including teacher-student interaction, classroom equipment, classroom management, classroom adaptability, teaching skills, classroom atmosphere, and feedback (*p* > 0.05 for all).

**Table 5:** Nonparametric Statistical Analysis Table for students of different grades.

	Grade Median (*P*_25_, *P*_75_)			Kruskal-Wallis H	*p*
	7.0 (*n* = 71)	8.0 (*n* = 70)	9.0 (*n* = 105)		
Overall	3.390(2.8,3.9)	3.410(3.0,3.9)	3.440(2.9,3.8)	0.338	0.844
Teacher-student interaction	3.430(2.4,4.3)	3.570(2.5,4.1)	3.570(2.6,4.1)	0.278	0.870
Classroom equipment	3.400(2.2,4.4)	3.800(2.8,4.5)	3.600(2.8,4.2)	1.157	0.561
Classroom management	3.600(2.6,4.6)	3.600(3.0,4.4)	3.400(2.7,4.3)	0.759	0.684
Classroom adaptability	3.600(2.6,4.6)	3.600(3.0,4.5)	3.600(2.8,4.4)	1.472	0.479
Teaching skills	3.670(2.3,4.5)	3.330(2.7,4.4)	3.500(2.7,4.2)	0.166	0.920
Classroom atmosphere	3.000(2.0,4.0)	3.250(2.3,4.1)	3.130(2.2,3.9)	0.887	0.642
Feedback	3.400(2.4,4.0)	3.000(2.2,3.8)	3.000(2.2,4.0)	0.971	0.615

*p < 0.05; **p < 0.01.

The responses from three interviewed students are provided to corroborate the conclusion: Student 1: “From what I have seen, whether it is seventh, eighth, or ninth graders, they all seem to have similar perceptions of our music teachers’ interaction decision-making levels. I have not noticed any significant differences based on grade level.” Student 2: “I agree. Our teachers, regardless of the grade we’re in, seem to handle classroom situations effectively. They adjust their teaching styles and interact with students with the aim of facilitating learning. I have not observed any notable differences in teaching methods based on grade.” Student 3: “I have also noticed that whether it’s seventh, eighth, or ninth-grade teachers, they all make decisions in class that help us learn. They interact with students and create a positive learning environment. I have not seen any significant differences in their interaction styles based on grade.” These consistent responses highlight the uniformity in music teachers’ approaches, further validating the statistical results. Future studies could explore whether this consistency is a result of structured teacher development programs or collaborative lesson planning across grade levels.

(3) Music Study Experiences.

Table 6 shows that there is a significant difference in overall perception between students with and without music study experiences. The Overall dimension showed significant differences at the 0.01 level (*p* = 0.000 < 0.01). Specifically, the median for students without music study experiences (3.610) was significantly greater than the median for students with music study experiences (2.990).

**Table 6:** Students’ non-parametric statistics based on whether they have Music Study experiences.

	Music study experiences Median (*P*_25_, *P*_75_)		MannWhitney U	MannWhitney z	*p*
	No (*n* = 168)	Yes (*n* = 78)			
Overall	3.610(3.2,3.9)	2.990(2.7,3.2)	3485.500	−5.905	0.000**
Teacher-student interaction	3.860(3.0,4.3)	2.710(2.1,3.6)	3658.000	−5.579	0.000**
Classroom equipment	3.800(2.8,4.5)	3.500(2.6,4.0)	5817.500	−1.417	0.156
Classroom management	3.600(2.6,4.4)	3.500(2.8,4.2)	6528.500	−0.045	0.964
Classroom adaptability	3.600(2.6,4.4)	3.600(3.0,4.4)	5739.000	−1.570	0.116
Teaching skills	3.830(2.8,4.5)	2.670(2.0,3.5)	3540.500	−5.806	0.000**
Classroom atmosphere	3.500(2.4,4.3)	2.500(1.9,3.3)	4278.500	−4.381	0.000**
Feedback	3.400(2.4,4.0)	2.500(2.0,3.6)	4695.000	−3.581	0.000**

*p < 0.05; **p < 0.01.

Further analysis of the sub-dimensions, including Teacher-student interaction, Classroom equipment, Classroom management, Classroom adaptability, Teaching skills, Classroom atmosphere, and Feedback, reveals differences. As shown in the table, whether students have music study experiences does not significantly affect Classroom equipment, Classroom management, and Classroom adaptability (all *p* > 0.05). However, whether students have music study experiences significantly affects Teacher-student interaction, Teaching skills, Classroom atmosphere, and Feedback (all *p* < 0.05).

Students’ music study experiences significantly impact various aspects of teacher-student interactions at the 0.01 level, with non-music students reporting higher median scores in teacher-student interaction (3.860 vs. 2.710), teaching skills (3.830 vs. 2.670), classroom atmosphere (3.500 vs. 2.500), and feedback (3.400 vs. 2.500) compared to their peers with music study experiences.

The responses from three interviewed students are provided to corroborate the conclusion: Student 1: “It seems like students who have not studied music before have a different perspective on how teachers interact with us. They seem to appreciate the interaction more and feel that the teachers without music study experiences connect with us better.” Student 2: “I noticed that students with no music study experiences tend to have more positive feedback on how teachers handle interactions in the classroom. It is like they feel more engaged and understood by those teachers.” Student 3: “I agree. Students without music study experiences seem to think that teachers without music backgrounds are better at interacting with us in class. They find the atmosphere more conducive to learning.” These responses from the interviewed students align with the findings from Table 6, indicating that students with different music study experiences perceive significant differences in music teachers’ interaction decision-making levels.

The findings indicate that students generally perceive their music teachers’ decision-making levels in classroom interactions positively. The overall mean score was 4.2, with a standard deviation of 0.7. Significant differences were observed across different grades and between students with and without music study experience. Second-grade students rated their teachers the highest, and students with music study experience had more favourable perceptions than did those without music study experience.

## Discussion

### Question 1

This analysis aligns with existing research, demonstrating that students hold a positive attitude toward teachers’ decision-making in classroom interaction, with an overall level of acceptance slightly above average. Students exhibit a high level of satisfaction with teacher interactions in the classroom, which is consistent with previous research^14,17^. For example, many students noted that teachers’ adaptive responses to their needs during lessons made them feel valued and more motivated to participate. Similarly, findings indicate a positive perception of teachers’ classroom teaching effectiveness, further supporting the idea that students thrive in environments where they feel their voices are heard and their learning styles are accommodated^21^.

During the student interviews, the participants expressed their appreciation for teachers who demonstrated adaptability and flexibility in the classroom. For example, Student 1 mentioned, “I like it when our music teacher is flexible with our learning pace. It makes me feel more comfortable asking questions and exploring new concepts.” This sentiment resonates with findings highlighting the positive impact of teacher adaptability on student satisfaction^22^.

Further analysis reveals that while students rate teachers highest in terms of classroom adaptability, they score lower in terms of classroom atmosphere and feedback. These quantitative findings are supported by insights from student interviews. For example, Student 2 mentioned, “Sometimes, the classroom atmosphere feels a bit dull, especially when there’s not much interaction among students. It would be great if our teacher could incorporate more group activities to liven up the class.” This echoes the importance of the classroom atmosphere highlighted and emphasizes the need for improvement in this aspect^23^.

Additionally, the students expressed a desire for more personalized and constructive feedback from their teachers. Student 3 stated, “I think feedback is crucial for our growth as musicians, but sometimes it feels a bit generic. It would be helpful if our teacher could provide more specific feedback tailored to individual needs.” This aligns with the findings underscoring the importance of timely and specific feedback in enhancing student learning and growth^24^. The desire for tailored feedback suggests that students are not only seeking validation but also constructive guidance that can help them navigate their musical development more effectively.

To address these issues, teachers in rural areas could incorporate more group activities and offer feedback that is individualized and timely. Such changes enhance student engagement and satisfaction, ultimately improving learning outcomes. These insights provide actionable guidance for educators to refine their teaching practices.

### Question 2

Although Research Question 2 examines students’ perceptions of teachers’ decision-making levels, it encompasses three key variables: gender, grade level, and music study experience. To ensure a clearer and more structured discussion, this section is divided into three corresponding parts. This approach allows for a more precise interpretation of the findings, ensuring that the impact of each variable is thoroughly analyzed.

(1) Gender.

According to the analysis, gender does not significantly influence the perception of teachers’ classroom interaction decision-making levels among middle school students. Specifically, there were no significant differences between the male and female student samples in terms of overall evaluation or in dimensions such as classroom equipment, classroom management, classroom adaptability, teaching skills, classroom atmosphere, and feedback. This finding aligns with previous research. For example, research has revealed no significant gender differences in the perceptions of teachers’ classroom interaction decision-making levels in music education, indicating similar cognitive attitudes toward teaching among both male and female students^5,6^.

During the student interviews, the participants provided additional insights into their perceptions of teacher’s classroom interaction decision-making levels. For example, Student 1 stated, “I do not think gender plays a significant role in how our music teacher makes decisions during class. Whether it is a male or female teacher, what matters most is their ability to engage with students and create a positive learning environment.” This sentiment was echoed by Student 2, who mentioned, “I have not noticed any differences in how male and female teachers interact with us during class. It is more about their teaching style and approach than their gender.”

Furthermore, students highlighted the importance of individual characteristics and teaching style over gender when evaluating teacher’s classroom interaction. Student 3 stated, “I think it is more about the teacher’s personality and teaching style than their gender. Some teachers, regardless of gender, are more interactive and engaging in class, while others may be more reserved. That’s what influences our perception of classroom interaction.” This comment underscores a critical takeaway: effective teaching hinges on personal attributes and pedagogical strategies rather than gender identity^10^.

These insights suggest that creating a positive, interactive classroom environment is key for all educators, regardless of gender. The findings highlight the importance of professional development in teaching skills and engagement strategies. By focusing on teaching effectiveness, educators can better meet the diverse needs of their students and improve their overall educational experience.

(2) Grade Level.

According to our analysis results, it is shown that there are no significant differences in the perceptions of teachers’ classroom decision-making levels among student samples of different grades. This finding is consistent with previous research. For instance, researchers have found no significant differences in teaching evaluations among different grades in education, suggesting a shared understanding of effective teaching practices among students^24–26^.

During the student interviews, the participants shared their perspectives on how their grade levels may influence their perceptions of teacher’s classroom decision-making levels. For example, Student 1 mentioned, “I have not noticed any differences in how our music teacher makes decisions based on our grade levels. Whether we’re in seventh or eighth grade, our teacher treats us all the same and makes decisions that are relevant to our learning.” This observation underscores the idea that a teacher’s equitable treatment of students can foster a sense of inclusivity and consistency in classroom management, regardless of grade. This sentiment was echoed by Student 2, who stated, “I do not think our grade levels affect how we perceive our teacher’s decision-making. It’s more about the teacher’s teaching style and approach.” This emphasizes that the quality of teaching, characterized by adaptability and engagement, is more influential than the students’ grade levels when it comes to their perceptions of decision-making.

Furthermore, students discussed other factors that may influence their perceptions of teachers’ classroom decision-making, in addition to grade level. Student 3 mentioned, “I think individual differences among students play a bigger role in how we perceive our teacher’s decisions. Some students may prefer a more structured approach, while others prefer more flexibility. It is not necessarily about grade levels but about what works best for each student.” This highlights the importance of recognizing diverse learning preferences and individual needs, suggesting that personalized teaching strategies can enhance student satisfaction and engagement more effectively than a one-size-fits-all approach at the grade level.

Additionally, the findings suggest that the effectiveness of classroom decision-making is more closely related to the teacher’s ability to connect with students and adapt to their varying needs, rather than to the students’ progression through grades. As such, educators may benefit from focusing on developing inclusive and flexible teaching practices that accommodate diverse student preferences, ensuring that all students feel valued and supported in their learning environment^27,28^.

Ultimately, the findings suggest that the effectiveness of classroom decision-making depends more on a teacher’s ability to connect with students and adapt to their needs than on students’ grade levels. Teachers should focus on developing flexible and inclusive teaching practices that recognize individual learning styles. By doing so, they can foster an engaging and supportive classroom environment, enhancing learning outcomes for all students, regardless of their grade level.

(3) Music Study Experiences.

The analysis results indicate significant differences in overall perception between students with and without music study experiences. Overall, the level of significance is 0.01, with the median score of students without music study experiences being significantly higher than that of students with music study experiences. This finding is consistent with previous research, suggesting a consistent pattern of the impact of music study experiences on perceptual cognition in educational settings^5,6^.

Further detailed analysis of the subscales revealed that the presence or absence of music study experiences significantly influences teacher-student interaction, teaching skills, the classroom atmosphere, and feedback (*p *< 0.05). Specifically, compared with students with music study experiences, students without music study experiences scored significantly higher in teacher-student interaction, teaching skills, classroom atmosphere, and feedback. These findings corroborate studies conducted by some studies^3,5,26^. Similar differences between students with and without music study backgrounds were observed in teacher-student interaction, teaching skills, and the classroom atmosphere. Research has emphasized the importance of recognizing the impact of students’ diverse learning experiences on shaping classroom dynamics and teaching quality^29^.

The differences in how students evaluate teachers’ decision-making levels may stem from multiple factors. Firstly, differences in cognitive perspectives significantly influence their evaluations. Students with music study experience typically possess musical knowledge and skills, leading to higher expectations regarding teacher interactions and classroom environments, with a greater emphasis on professional depth^5^. In contrast, students without study experiences tend to focus more on overall interaction and the atmosphere. During the interviews, the students shared their perspectives on these differences. For instance, Student 1 expressed, “I think because I have learned music, I notice how my teacher engages with us. I expect more depth and professionalism in their decisions.” In contrast, Student 2, who has no music experience, stated, “I just enjoy the class. I do not think about it too much; I just want to have fun and participate.” This highlights how students’ backgrounds shape their perceptions.

Secondly, the differences in learning motivation are also noteworthy. Students without music study experiences may find music learning novel and exciting, positively evaluating the interactive atmosphere created by teachers, whereas experienced students, who have undergone formal education, tend to have higher teaching standards and stricter evaluations^8^. Student 3 noted, “For me, music class is just different from other classes; I feel it is fun and engaging, so I think the teacher is doing well.” This sentiment indicates that novelty can enhance positive evaluations.

Additionally, variations in classroom participation also contribute to differences in evaluation. Students with music study experiences are generally more active and independent in participating in classes and assessing teaching from a professional perspective, whereas those without such experience may rely more on teacher guidance^15^. Finally, cultural backgrounds and social expectations play important roles. Students with music study experiences may come from families that value music education more highly, resulting in higher expectations of teachers, whereas students without such experience may hold a more positive view of any form of music instruction^18,27^.

Therefore, this study underscores the significant influence of music study experiences on students’ perceptions of classroom interaction, teaching effectiveness, and overall classroom atmosphere. Educators should recognize and adapt to the diverse learning backgrounds of students to optimize educational outcomes and enhance the classroom experience for all.

### Implementation

Effective music education requires a dynamic and responsive teaching approach, where educators actively adapt to students’ needs, fostering an engaging and interactive classroom environment. Research suggests that student-centered learning enhances both motivation and comprehension^26^. The following strategies highlight keyways to improve classroom interaction, feedback, atmosphere, differentiated instruction, and cultural integration in music education.

In classroom interactions, teachers can adjust the teaching pace and content based on student feedback, ensuring alignment with students’ comprehension levels and learning needs. Strategies such as group discussions and quick polls allow real-time assessment of understanding, enabling immediate clarification and deeper exploration of concepts^9^. Encouraging structured questioning and peer discussions fosters an interactive learning environment, enhancing student engagement and active participation^6,27,29^.

To ensure meaningful feedback, a structured progress-tracking system can help monitor individual development and inform instructional adjustments. Regular one-on-one check-ins provide students with dedicated opportunities to discuss study experiences, express concerns, and receive targeted support, reinforcing motivation and accountability^12^.

Creating a positive classroom atmosphere is crucial for sustaining engagement in music education. Interactive activities such as music games, role-playing, and group performances encourage participation and deepen conceptual understanding^13^. Collaborative projects, such as composing simple melodies or engaging in rhythmic exercises, foster creativity and active learning^14^. Displaying student work, organizing music sharing moments, and hosting informal performances contribute to a sense of achievement and community, strengthening student confidence and classroom culture^5^.

Differentiated instruction, particularly grouping students based on musical background, enhances learning outcomes. Advanced students benefit from tasks such as music analysis, composition, and improvisation, while beginners develop foundational skills, including rhythm, pitch recognition, and basic instrumental techniques^4^. Formative assessments, including self-reflection and peer feedback, play a crucial role in guiding instructional adaptations^3,28^.

Integrating local musical traditions with global styles enriches students’ cultural awareness and broadens their musical perspectives. Exposure to traditional folk songs, indigenous instruments, and regional music practices fosters connections with cultural heritage while promoting an appreciation for diverse musical expressions^15^. Hands-on experiences, such as field trips to performances or artist workshops, enhance students’ understanding of music’s societal significance^16^. Organizing a cross-cultural music festival or collaborative projects with musicians from diverse backgrounds can further support global music literacy and intercultural competence^23^.

By applying these strategies, teachers can create a more engaging and effective music education experience tailored to student needs.

### Research limitations

This study offers valuable insights into the classroom decision-making levels of rural middle school music teachers, but it has several limitations. First, the small sample size from a single rural school limits the generalizability of the findings. Expanding the sample across different regions could enhance representativeness. Second, resource constraints, such as limited music education equipment, may have influenced student perceptions and classroom interactions. Additionally, variations in students’ backgrounds, including prior music experience and family support, were not deeply analyzed, potentially affecting results. Lastly, reliance on self-reported data may introduce bias; future research could incorporate classroom observations and teacher interviews for a more comprehensive perspective.

## Conclusion

This study provides valuable insights into the decision-making levels of rural middle school music teachers from the students’ perspective. It emphasizes the importance of teacher-student interaction, classroom management, adaptability, teaching skills, and feedback in shaping learning experiences. The findings highlight challenges in rural education, such as limited resources, which impact students’ perceptions of teaching effectiveness. The study suggests that teachers in rural areas should prioritize flexibility, incorporate local cultural elements, and offer personalized feedback to enhance engagement. For rural teachers, strategies such as peer-led activities and fostering a collaborative classroom environment can be low-cost ways to improve the teaching experience. Further research is needed, particularly in expanding sample sizes and exploring student background differences. This research contributes to the understanding of rural music education, providing practical recommendations for educators and policymakers. Future studies should continue to explore sustainable strategies for rural education to meet students’ diverse needs.

## Electronic supplementary material

Below is the link to the electronic supplementary material.

Supplementary Material 1

## Data Availability

The data that support the findings of this study are available on request from the corresponding author.
