# Supplementary material for: Exploring rural middle school music teachers’ classroom interaction decision-making levels: A student’s perspective
Source: Sci Rep. 2025 Feb 17;15:5798. doi: 10.1038/s41598-025-90503-4 (PMC11832737; doi:10.1038/s41598-025-90503-4)
Supplement: Supplementary file 1 — Supplementary Material 1 [file 41598_2025_90503_MOESM1_ESM.docx]

**Questionnaire**

Dear students：

Thank you for participating in this questionnaire survey, aimed at understanding students' perceptions of the teaching decision-making levels of novice music teachers from a student perspective. Your valuable insights will greatly contribute to our research. Your responses will be kept strictly confidential and used only for research analysis. Please note that your participation requires your understanding and acceptance of informed consent before you can respond.

The questionnaire is divided into two parts. The first part consists of demographic questions. The second part includes 21 questions related to 7 influencing factors, to be answered using the Likert five-point scale: 1 - Strongly Disagree, 2 - Disagree, 3 - Neutral, 4 - Agree, 5 - Strongly Agree.

Section A:

The first section is for demographic issues: for example, gender, grades, and with or without music study experiences data to collect the specific information of students, a total of 3 questions.

| No | Questions | Options |
| --- | --- | --- |
| 1 | Gender | A: Male  B: Female |
| 2 | Grades | A: 7  B: 8  C: 9 |
|  | Music study experiences | A: With  B: Without |

Table 3.4.1 Demographic information

Section B:

The second part includes 21 questions related to 7 influencing factors, to be answered using the Likert five-point scale: 1 - Strongly Disagree, 2 - Disagree, 3 - Neutral, 4 - Agree, 5 - Strongly Agree.

| No | Items | 1 | 2 | 3 | 4 | 5 |
| --- | --- | --- | --- | --- | --- | --- |
| 1 | Teachers provide me with ample opportunities to participate in classroom interactions, such as selecting course projects or extracurricular activities. |  |  |  |  |  |
| 2 | Throughout my learning process, teachers frequently interact with me. |  |  |  |  |  |
| 3 | I believe teachers in music classes can consider each student's individual circumstances fully. |  |  |  |  |  |
| 4 | I believe teachers effectively maintain discipline in music classroom management. |  |  |  |  |  |
| 5 | I believe teachers effectively allocate time during class in music classrooms. |  |  |  |  |  |
| 6 | I believe music teachers' classroom management contributes to creating a positive learning environment. |  |  |  |  |  |
| 7 | I believe teachers successfully create a positive, supportive atmosphere in music classrooms. |  |  |  |  |  |
| 8 | I am willing to participate in music classroom activities and discussions in this musical environment. |  |  |  |  |  |
| 9 | I believe music teachers can adjust teaching strategies to meet the needs of different students. |  |  |  |  |  |
| 10 | I believe music teachers can quickly adapt to new teaching methods. |  |  |  |  |  |
| 11 | I believe teachers can adjust teaching methods based on students' actual learning situations. |  |  |  |  |  |
| 12 | I believe teachers can vividly explain music knowledge and capture my attention. |  |  |  |  |  |
| 13 | I believe teachers can skillfully use various music teaching methods to enhance my music learning. |  |  |  |  |  |
| 14 | I believe music teachers can effectively organize and design classroom activities. |  |  |  |  |  |
| 15 | I believe music teachers can fully utilize classroom equipment to support and enrich music classroom teaching. |  |  |  |  |  |
| 16 | I believe there is adequate equipment in the classroom to assist music teachers in their teaching. |  |  |  |  |  |
| 17 | I believe teachers provide clear and engaging music teaching experiences when using instructional equipment. |  |  |  |  |  |
| 18 | I believe music teachers can promptly devise alternative solutions when equipment malfunctions. |  |  |  |  |  |
| 19 | I believe music teachers provide me with timely and targeted feedback to help me progress in my music learning. |  |  |  |  |  |
| 20 | I believe the feedback provided by teachers is clear and easy to understand. |  |  |  |  |  |
| 21 | I believe that teachers' feedback on my learning helps me better apply the music knowledge I have acquired. |  |  |  |  |  |
